# Deep learning reconstruction improves radiomics feature stability and discriminative power in abdominal CT imaging: a phantom study

**DOI:** 10.1007/s00330-022-08592-y

**Published:** 2022-02-16

**Authors:** Florian Michallek, Ulrich Genske, Stefan Markus Niehues, Bernd Hamm, Paul Jahnke

**Affiliations:** 1 Department of Radiology, Charité – Universitätsmedizin Berlin, corporate member of Freie Universität Berlin, Humboldt-Universität zu Berlin, and Berlin Institute of Health, Charitéplatz 1, 10117 Berlin, Germany; 2 Data Analytics and Computational Statistics, Hasso Plattner Institute, Digital Engineering Faculty, University of Potsdam, 14482 Potsdam, Germany; 3 Berlin Institute of Health (BIH), Anna-Louisa-Karsch-Str. 2, 10178 Berlin, Germany

**Keywords:** Tomography, X-ray computed, Phantoms, imaging, Liver neoplasms, Algorithms, Reproducibility of results

## Abstract

**Objectives:**

To compare image quality of deep learning reconstruction (AiCE) for radiomics feature extraction with filtered back projection (FBP), hybrid iterative reconstruction (AIDR 3D), and model-based iterative reconstruction (FIRST).

**Methods:**

Effects of image reconstruction on radiomics features were investigated using a phantom that realistically mimicked a 65-year-old patient’s abdomen with hepatic metastases. The phantom was scanned at 18 doses from 0.2 to 4 mGy, with 20 repeated scans per dose. Images were reconstructed with FBP, AIDR 3D, FIRST, and AiCE. Ninety-three radiomics features were extracted from 24 regions of interest, which were evenly distributed across three tissue classes: normal liver, metastatic core, and metastatic rim. Features were analyzed in terms of their consistent characterization of tissues within the same image (intraclass correlation coefficient ≥ 0.75), discriminative power (Kruskal-Wallis test *p* value < 0.05), and repeatability (overall concordance correlation coefficient ≥ 0.75).

**Results:**

The median fraction of consistent features across all doses was 6%, 8%, 6%, and 22% with FBP, AIDR 3D, FIRST, and AiCE, respectively. Adequate discriminative power was achieved by 48%, 82%, 84%, and 92% of features, and 52%, 20%, 17%, and 39% of features were repeatable, respectively. Only 5% of features combined consistency, discriminative power, and repeatability with FBP, AIDR 3D, and FIRST versus 13% with AiCE at doses above 1 mGy and 17% at doses ≥ 3 mGy. AiCE was the only reconstruction technique that enabled extraction of higher-order features.

**Conclusions:**

AiCE more than doubled the yield of radiomics features at doses typically used clinically. Inconsistent tissue characterization within CT images contributes significantly to the poor stability of radiomics features.

**Key Points:**

• *Image quality of CT images reconstructed with filtered back projection and iterative methods is inadequate for the majority of radiomics features due to inconsistent tissue characterization, low discriminative power, or low repeatability.*

• *Deep learning reconstruction enhances image quality for radiomics and more than doubled the feature yield at doses that are typically used in clinical CT imaging.*

• *Image reconstruction algorithms can optimize image quality for more reliable quantification of tissues in CT images.*

**Supplementary Information:**

The online version contains supplementary material available at 10.1007/s00330-022-08592-y.

## Introduction

Radiomics uses quantitative features extracted from computed tomography (CT) images to build predictive models for improved diagnosis, prognosis, and therapy of cancer [1, 2]. However, inadequate robustness towards clinical image quality limits the development and application of radiomics [3]. Influences resulting from the imaging process affect feature extraction to the point of making radiomics features nonreproducible and excluding most features from disease assessment [4]. It is therefore of interest to better understand image quality requirements for radiomics and to identify imaging techniques that increase the yield of reliable features.

Image reconstruction algorithms are of particular interest in that they determine how the photon signal is processed to generate images that accurately display tumor properties. Filtered back projection (FBP) and iterative methods, which were used in most radiomics studies, impair the stability of radiomics features [5, 6]. Recently introduced deep learning reconstruction was reported to control noise, which is particularly abundant with FBP, and to maintain noise texture, which is a limitation of iterative reconstruction [7, 8]. In light of these improvements, deep learning reconstruction may allow more reliable quantification of tissues for extraction of radiomics features.

Previous work investigated image reconstruction and feature stability in phantoms or in patients [4, 9]. Phantoms have the advantage of enabling repeated scans, but frequently provide simplified textures that may not adequately reflect the complexity of human tissues. By contrast, patients have authentic tissue texture, but cannot be scanned repeatedly, and patient examinations involve greater uncertainty regarding comparability of the investigated tissue ground truth. While several studies reported feature stability when switching between reconstructions [4–6, 10, 11], this approach does not consider differences in image quality that may fundamentally limit application of particular reconstruction algorithms for radiomics. Moreover, feature stability was previously reported across different images, but little is known about stability within the same image for characterizing tissues at different locations in the scan field.

Given these limitations, the present work used advances in 3D printing of realistic textured radiomics phantoms [12, 13] to create a phantom simulating a patient with hepatic metastases. The phantom was used to independently evaluate four generations of reconstruction algorithms for fundamental differences in image quality for radiomics. Feature analysis was expanded to include consistency, a measure of feature stability within the same CT image, discriminative power, and repeatability. The work was motivated by the hypothesis that deep learning reconstruction improves feature stability and discriminative power compared to reconstruction methods previously used for radiomics. Based on this assumption, the aim was to compare the image quality of deep learning reconstruction for radiomics feature extraction with FBP, hybrid iterative reconstruction, and model-based iterative reconstruction.

## Methods

The institutional ethics committee approved the study and waived informed consent.

### Phantom

**Fig. 1:**
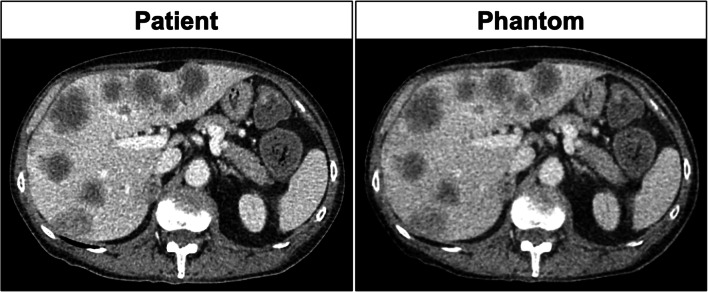
Comparison of CT images of the patient (left) and of the phantom (right). Both images were reconstructed with 1 mm slice thickness and are displayed with the same window settings

A CT image of a 65-year-old patient with rectal cancer and hepatic metastases was retrospectively selected from our clinical database. The CT image was used as a template for manufacturing a 1-cm-thick abdominal phantom using radiopaque 3D printing, which involves inkjet printing with iodine-doped ink on paper followed by assembling the printed paper sheets to create mechanically stable phantoms [14, 15]. Assembly involved a three-step process consisting of stacking, gluing, and cutting every paper sheet to the patient’s shape. A polyethylene film (8 g/m^2^) served as thermoplastic adhesive, replacing the toner used in previous work [15]. The technique enables the manufacture of realistic textured phantoms and was used previously to create phantoms for the analysis of radiomics features [12, 13]. The phantom used here consisted of repeated prints of the same template image, which means that all acquired phantom images displayed the same abdominal slice of the patient. Figure 1 shows the patient’s CT image and the phantom used here.

### Image acquisition

The phantom was scanned on a Canon Aquilion One Genesis CT scanner (Canon Medical Systems). The tube voltage was 100 kVp, the rotation time was 0.5 s, and the pitch was 0.813. Eighteen different tube currents were used, and 20 repeated acquisitions were performed per tube current. Table 1 summarizes the tube currents and the resulting CT dose indices (CTDI_vol_). Images were reconstructed with 1 mm slice thickness and 0.8 mm increment using FBP with a soft tissue kernel (FC08) and the manufacturer’s implementation of hybrid iterative reconstruction, model-based iterative reconstruction, and deep learning reconstruction: Adaptive Iterative Dose Reduction 3D (AIDR 3D), Forward projected model-based Iterative Reconstruction SoluTion (FIRST), and Advanced intelligent Clear-IQ Engine (AiCE). A total of 1440 datasets were thus generated (18 tube currents × 20 repetitions × 4 reconstruction methods).

**Table 1:** Tube currents and resulting computed tomography dose indices (CTDI_vol_) used for image acquisition

Tube current (mA)	10	20	30	40	50	60	70	80	90	100	110	120	130	140	150	160	180	200
CTDI_vol_ (mGy)	0.2	0.4	0.6	0.8	1	1.2	1.4	1.6	1.8	2	2.2	2.4	2.6	2.8	3	3.2	3.6	4

### Radiomics feature extraction

**Fig. 2:**
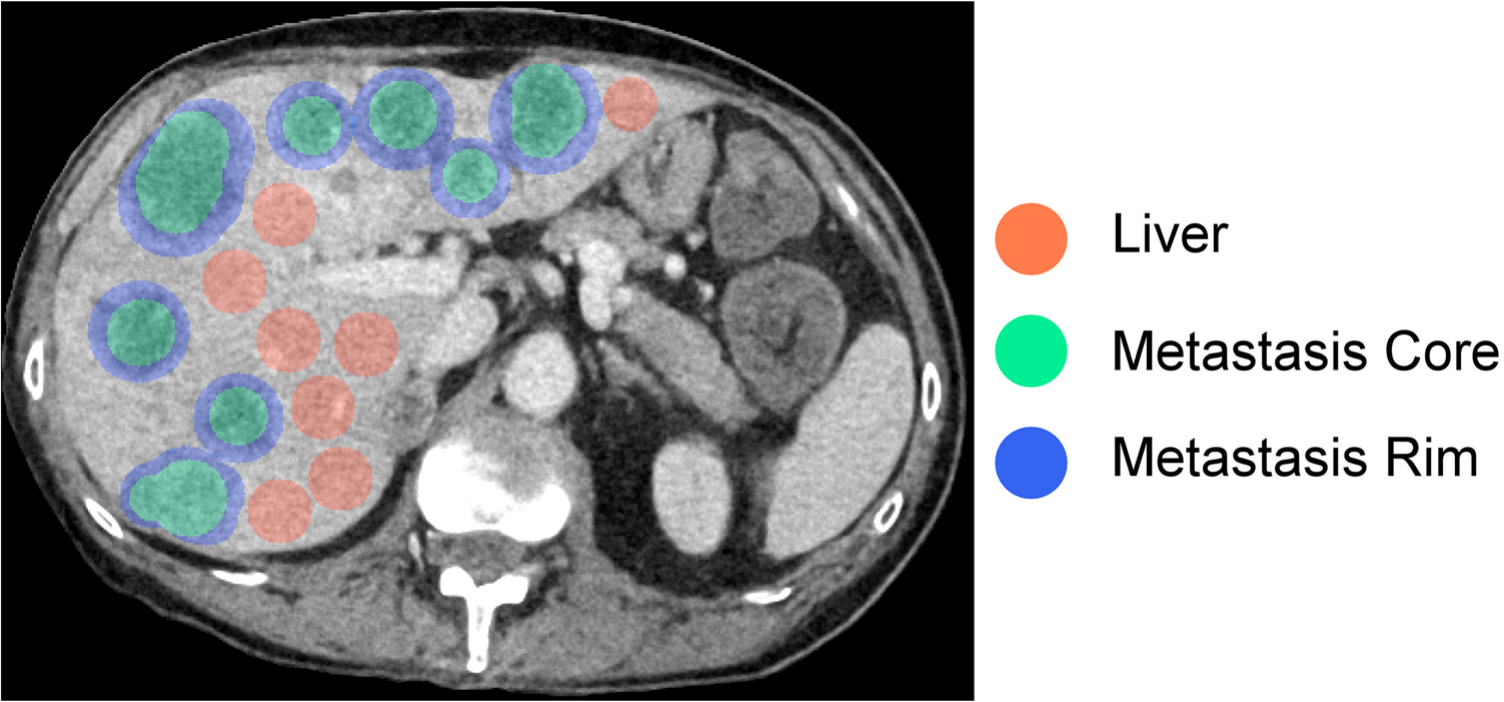
Region of interest (ROI) placement for radiomics feature extraction. Three tissue classes (liver, metastatic core, and metastatic rim) were analyzed using eight ROIs per tissue class

Four adjacent images from the phantom center were extracted from each of the 1440 datasets and treated as image volumes in the subsequent analysis. The same 24 regions of interest (ROIs) were placed in the same positions in all images: 8 in the liver parenchyma, 8 in the metastatic core, and 8 in the metastatic rim (Fig. 2). Each of the 24 ROIs was subjected to radiomics analysis using 93 features from the following feature groups: 18 first-order features, 24 gray-level co-occurrence matrix features (GLCM), 14 gray-level dependence matrix features (GLDM), 16 gray-level run length matrix features (GLRLM), 16 gray-level size zone matrix features (GLSZM), and 5 neighboring gray-tone difference matrix features (NGTDM). Shape features were not analyzed, since ROI shapes were not varied, and an investigation of segmentation variability was not the aim of this study. For extraction of radiomics features, we used the previously validated PyRadiomics package (version 2.2.0) with standard settings as recommended by the authors of the package [16]. Specifically, the standard fixed bin width of 25 was used, and features were calculated as implemented without code modification. A detailed definition of all features is available in the PyRadiomics documentation [17]. We did not apply prefiltering or any other image manipulation prior to feature extraction.

### Statistical analysis

Radiomics features extracted from the three tissue classes investigated (liver parenchyma, metastatic core, and metastatic rim) were analyzed with regard to three outcome parameters: (1) consistency, which means the agreement between features extracted from the same tissue class in different image positions; (2) discriminative power, which means the ability of features to distinguish between the three tissue classes; and (3) repeatability, which means the agreement between features in scan-rescan experiments.

Feature consistency was assessed using the intraclass correlation coefficient (ICC) [18]. The ICC was calculated across the three investigated tissue classes using feature values extracted from eight ROIs per tissue class, dose, and image reconstruction. A two-way mixed, single score was calculated, corresponding to ICC type (3,1) according to Shrout and Fleiss [19], and the median from 20 repeated acquisitions was calculated. The discriminative power of features was evaluated using the Kruskal-Wallis test. Tissue classes were compared using feature values extracted from eight ROIs per tissue class, dose, and image reconstruction. The *p* value was adjusted for multiple comparisons using Bonferroni’s method. Repeatability was analyzed using the overall concordance correlation coefficient (OCCC). The OCCC is an extension of the twofold concordance correlation coefficient (CCC) that accounts for multiple comparisons [20]. The OCCC was calculated across 20 repeated acquisitions per dose and image reconstruction. The calculation was performed for each tissue class separately and then averaged.

The analysis of the ICC, Kruskal-Wallis test, and OCCC was based on acceptance criteria, which were defined as follows: results ≥ 0.75 were considered acceptable for the ICC and the OCCC. For the Kruskal-Wallis test, a *p* value < 0.05 in ≥ 95% of the 20 repeated acquisitions was considered significant in indicating that features sufficiently discriminated between tissue types. Features were further clustered according to these results in a group of features that were classified as robust by complying with the acceptance criteria for all three outcome parameters.

Correlation analysis of dose and feature yield was performed using Pearson correlation. Estimates are given as correlation coefficient *r* along with *p* values.

## Results

**Fig. 3:**
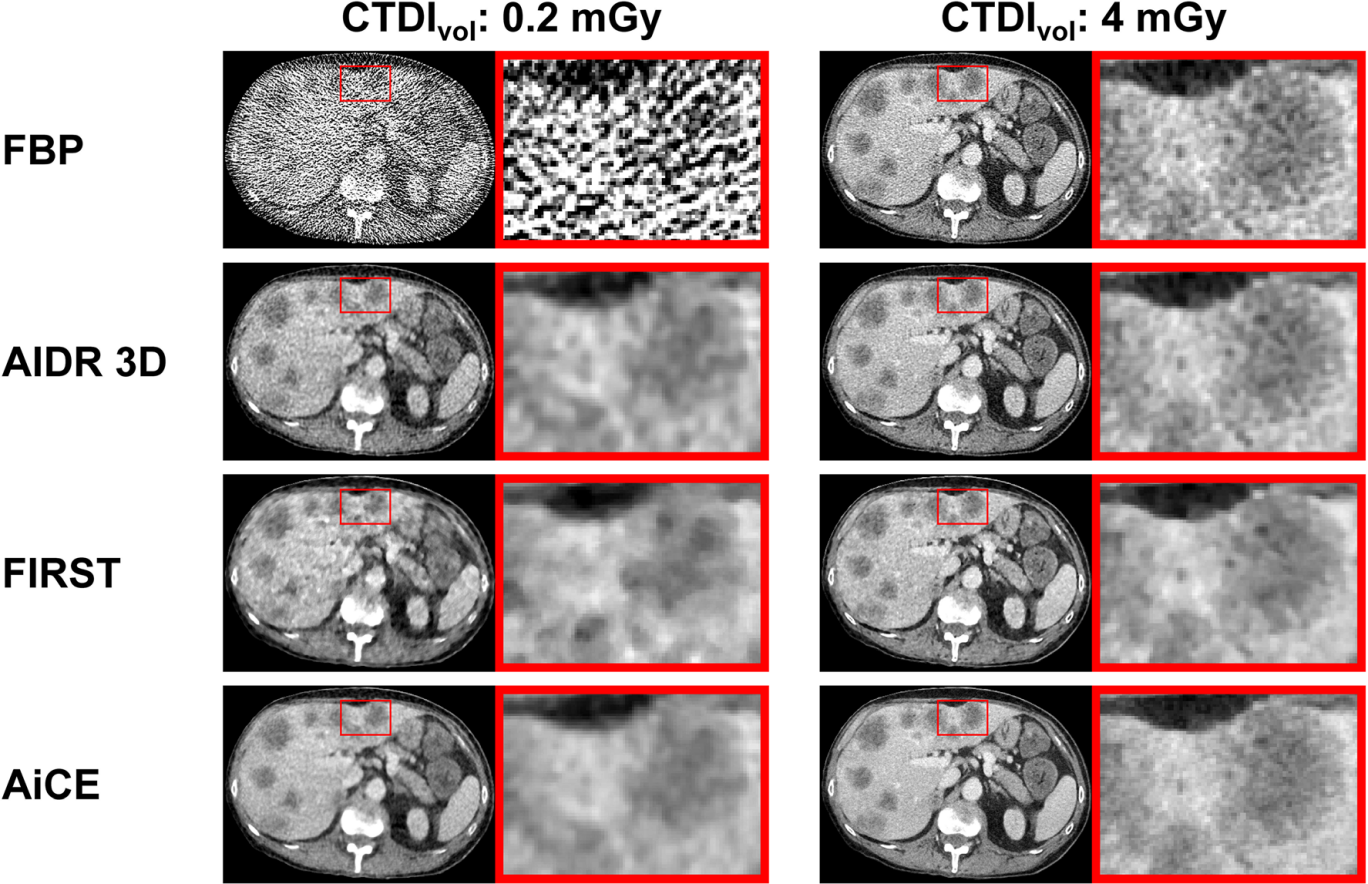
CT images acquired with 0.2 and 4 mGy and reconstructed with four different reconstruction algorithms. FBP is filtered back projection. AIDR 3D (Adaptive Iterative Dose Reduction 3D), FIRST (Forward projected model-based Iterative Reconstruction SoluTion), and AiCE (Advanced intelligent Clear-IQ Engine) are the manufacturer’s implementation of hybrid iterative reconstruction, model-based iterative reconstruction, and deep learning reconstruction, respectively

Figure 3 shows a series of CT images acquired with 0.2 and 4 mGy and reconstructed with FBP, AIDR 3D, FIRST, and AiCE. Images acquired with 0.2 mGy demonstrate the strong impact of noise at low doses with FBP reconstruction and the denoising power of AIDR 3D, FIRST, and AiCE. Furthermore, images shown in Fig. 3 illustrate how different reconstruction algorithms affect CT images and lesions contained herein both at lower and higher doses.

**Fig. 4:**
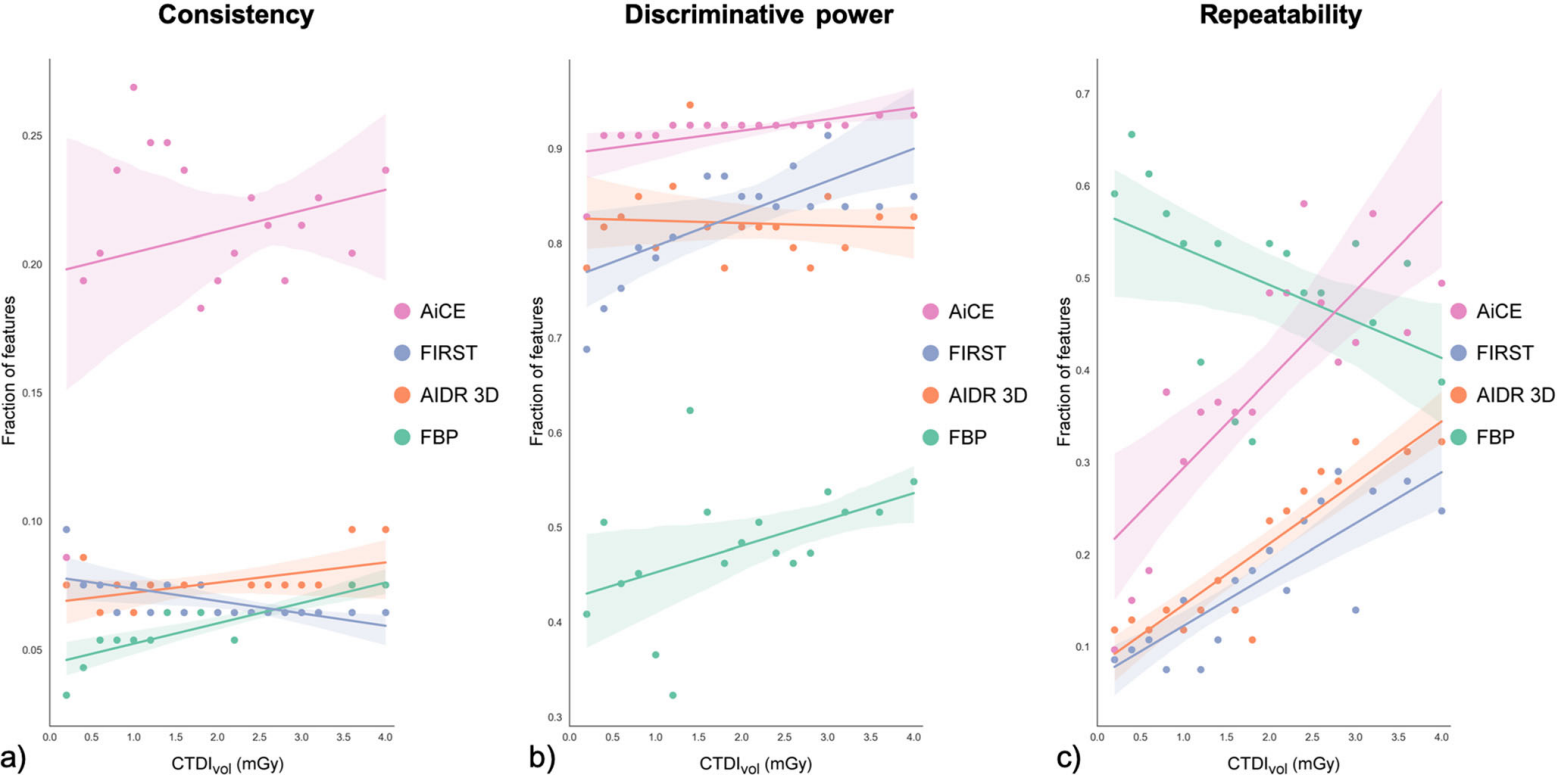
Fraction of radiomics features that yielded acceptable consistency (**a**), discriminative power (**b**), and repeatability (**c**) per dose and image reconstruction. Acceptance criteria were an intraclass correlation coefficient (ICC) ≥ 0.75 for consistency, a *p* value of the Kruskal-Wallis test < 0.05 in ≥ 95% of acquisitions for discriminative power, and an overall concordance coefficient (OCCC) ≥ 0.75 for repeatability. Colored shades accompanying the trend lines indicate 95% confidence intervals

Figure 4 presents, for each dose and reconstruction method, the fraction of radiomics features that achieved adequate consistency in characterizing tissues of the same tissue class at different locations in the scan field (ICC ≥ 0.75), adequate discriminative power in differentiating between tissues of different classes (*p* < 0.05 in ≥ 95% of repeated acquisitions), and adequate repeatability (OCCC ≥ 0.75). Detailed results are provided in suppl. figs. 1 to 3.

### Feature consistency

Images reconstructed with FBP and iterative methods severely affected the stability of radiomics features across different locations in CT images (Fig. 4a). Only 6% of features yielded consistent results with FBP (median across all doses, range 3 to 8%), 8% with AIDR 3D (range 6 to 10%), and 6% with FIRST (range 6 to 10%). Deep learning reconstruction improved image quality for consistent tissue quantification. With use of AiCE, consistent features increased to 22% (median, range 9 to 27%), with a marked decrease to 9% only at the lowest dose (0.2 mGy).

### Discriminative power

FBP reconstruction had a surprisingly strong impact on features in discriminating between tissues (Fig. 4b). Only 48% of features had adequate discriminative power (median, range 32 to 62%), and discriminative power remained low even at higher doses, at which humans can be expected to distinguish between tissues without much difficulty. Denoising reconstruction methods significantly improved the discriminative power of radiomics features. The median feature yield was 82% with AIDR 3D (range 77 to 95%) and 84% with FIRST (range 69 to 92%). Again, AiCE yielded superior results (median 92% of features, range 83 to 94%) with a decrease to 83% at 0.2 mGy.

### Feature repeatability

Feature stability across repeated acquisitions was highest with FBP (Fig. 4c). The median fraction of repeatable features was 52% (range 32 to 66%). However, FBP results deteriorated with dose (*r* = − 0.49, *p* = 0.04), and many features were repeatable especially at very low doses (e.g., 66% of features at 0.4 mGy). In contrast, repeatable features increased with dose in images reconstructed with AIDR 3D (*r* = 0.91, *p* < 0.001), FIRST (*r* = 0.84, *p* < 0.001), and AiCE (*r* = 0.81, *p* < 0.001). However, the overall yield of repeatable features was low with any of the iterative methods. The median was only 20% with AIDR 3D (range 11 to 32%) and 17% with FIRST (range 8 to 29%). Results increased to 39% with AiCE (median, range 10 to 58%), again with a marked decrease to below 18% at very low doses (≤ 0.6 mGy).

### Features deemed robust

**Fig. 5:**
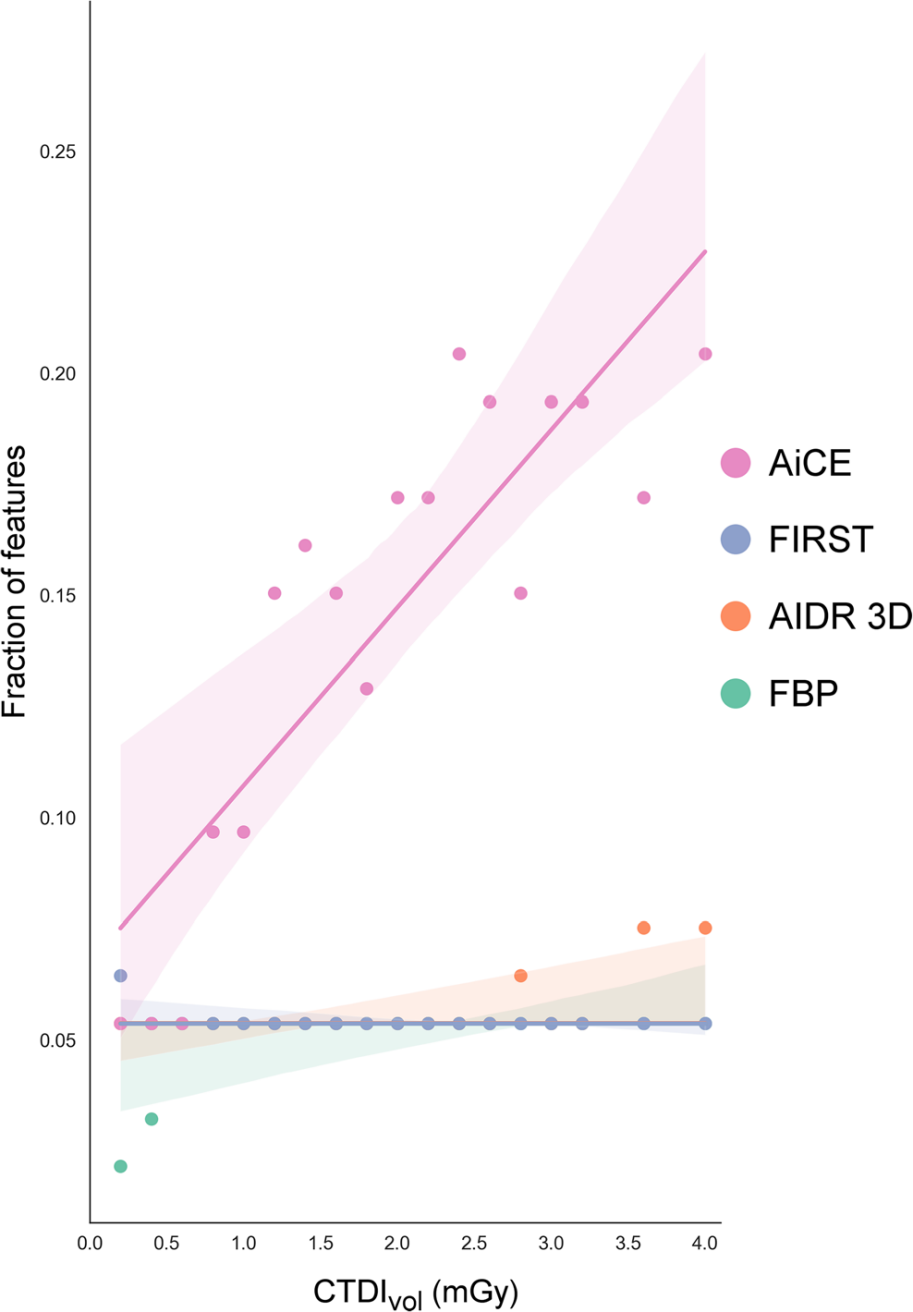
Fraction of radiomics features that were classified as robust by combining consistency, discriminative power, and repeatability per dose and image reconstruction. Colored shades accompanying the trend lines indicate 95% confidence intervals

Figure 5 summarizes, for each dose and reconstruction method, the fraction of radiomics features that were classified as robust by combining consistency, discriminative power, and repeatability. FBP, AIDR 3D, and FIRST each yielded poor results, which was due to the low discriminative power of features with FBP, low repeatability with AIDR 3D and FIRST, and low consistency of features with all three reconstruction methods. The median fraction of robust features was 5% for all three reconstructions; ranges were 2 to 5% for FBP, 5 to 8% for AIDR 3D, and 5 to 6% for FIRST. AiCE increased the feature yield to 16% (median, range 5 to 20%). Poor results of 5% at doses ≤ 0.6 mGy reflect the decrease in consistency, discriminative power, and repeatability at very low doses described in the previous sections. The number of robust features increased with dose in AiCE-reconstructed images (*r* = 0.86, *p* < 0.001) to 20% at 4 mGy.

**Fig. 6:**
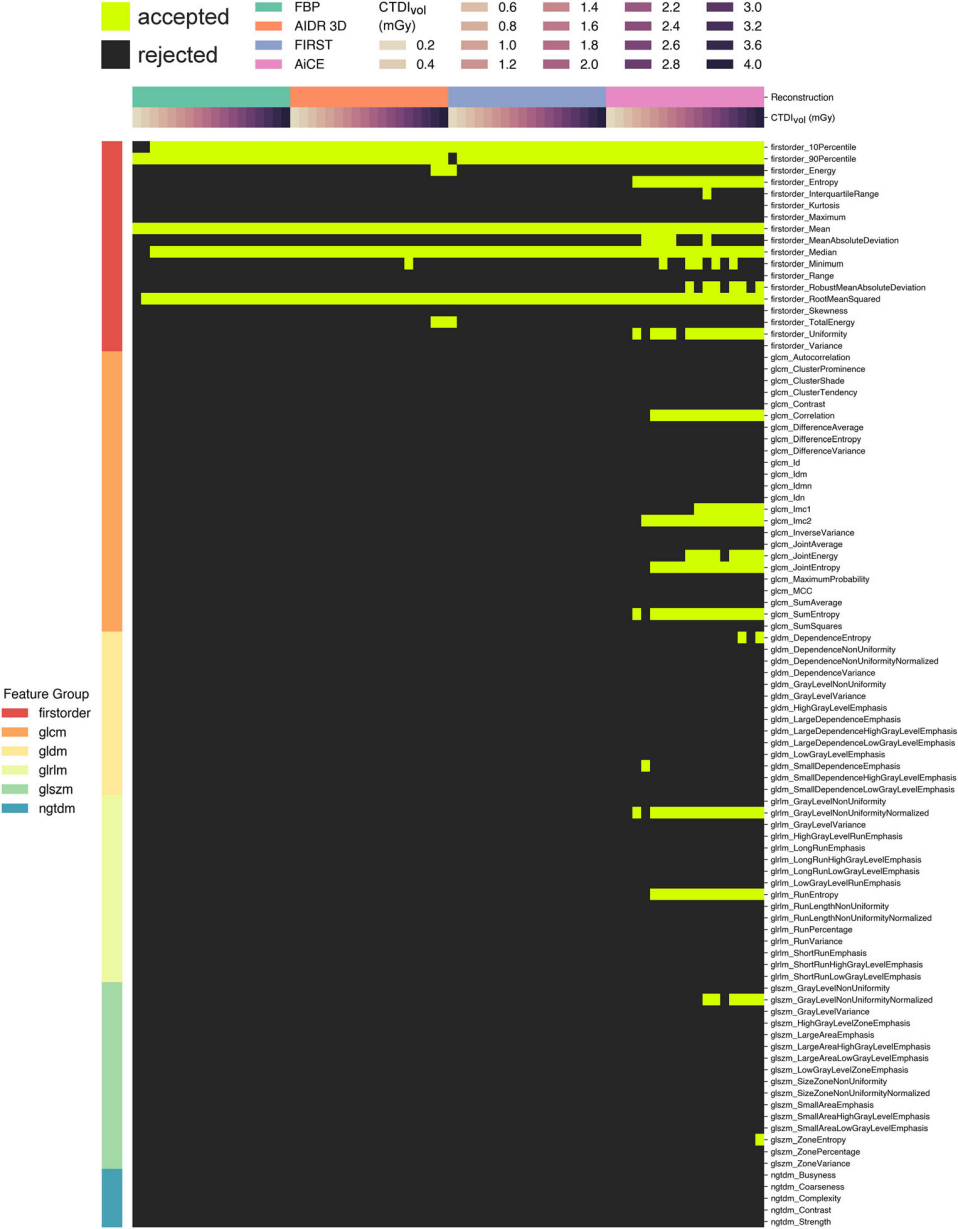
Individual presentation of radiomics features that were classified as robust. Light green squares indicate features that complied with the acceptance criteria for consistency, discriminative power, and repeatability per dose and image reconstruction

Figure 6 provides a detailed presentation of robust radiomics features per dose and reconstruction method. Only a few first-order features met the acceptance criteria with use of FBP, AIDR 3D, and FIRST. These features were robust largely independently of dose, with few exceptions at low doses for FBP and FIRST. AiCE significantly expanded the spectrum of robust features. In particular, several additional first-order, GLCM, and GLRLM features were robust with AiCE independently of dose, except for the lowest doses ≤ 1 mGy. Some first-order, GLCM, and GLSZM features were robust only at higher doses ≥ 3 mGy. There were also some features with variable robustness at similar dose levels, which suggests that these features may be more sensitive to slight variations in the acquisition mode. In a comparison of features that were robust independently of dose above 1 mGy, FBP, AIDR 3D, and FIRST each yielded 5/93 features versus 12/93 with AiCE. With AiCE, this number further increased to 16/93 at doses ≥ 3 mGy.

## Discussion

Image reconstruction severely affects the stability of radiomics features extracted from CT images. Here, we compared the image quality of deep learning reconstruction for radiomics feature extraction with filtered back projection, hybrid iterative reconstruction, and model-based iterative reconstruction at doses ranging from 0.2 to 4 mGy. We used a patient-mimicking phantom with hepatic metastases to analyze the discriminative power of features, feature stability across different positions in CT images, and feature stability in repeated acquisitions. At typical clinical doses above 1 mGy, only 5% of features combined discriminative power and stability within and across repeated acquisitions with FBP and iterative methods. Deep learning reconstruction enhanced image quality for radiomics feature extraction and more than doubled the feature yield to 13% at doses > 1 mGy and 17% at doses ≥ 3 mGy.

Poor feature stability across different images is a limitation of radiomics that has been reported in several studies investigating reproducibility [4–6, 9–13, 21–23]. Our experiments show that, even within the same acquisition and reconstruction, feature consistency, a metric of reproducibility within the same image, is low across identical tissue classes in different image positions. Inhomogeneous image quality, e.g., due to textured and nonstationary noise [24], thus fundamentally degrades feature extraction and contributes to the poor reproducibility of radiomics features. Deep learning reconstruction improves the consistent quantification of tissues in CT images, thus providing a better data basis for the extraction of more reliable radiomics features.

Filtered back projection and iterative reconstruction were involved in most previous developments of radiomics in computed tomography. FBP is a linear reconstruction algorithm, which enhanced feature stability across repeated acquisitions in our experiments. However, the negative dose correlation and high repeatability especially at low doses suggest that a significant part of the repeatability results was due to repetitive noise with limited value for actual tissue classification, an interpretation supported by the low discriminative power of FBP images. Iterative methods denoise images using nonlinear operations, which was essential for enhancing the discriminative power of radiomics features. However, this improvement came at the expense of impaired repeatability especially at low doses, at which strong denoising of iterative reconstruction alters the noise texture [25].

Deep learning reconstruction uses a deep learning neural network to enhance image quality and was reported to remove noise from signal without changing noise texture itself [7, 8]. Our results confirm the improvement in image quality for radiomics. Deep learning reconstruction improved all aspects of feature extraction and was the only method that produced adequate images for the extraction of higher-order features. This superiority was lost when raw data quality was too poor at very low doses. Conversely, higher doses improved the stability of some features, which adds to previous reports of dose effects on feature stability [4, 11]. The majority of features identified here, however, could be used largely independently of dose, showing that deep learning reconstruction provides a fairly robust data basis for feature extraction at doses typically used in clinical imaging.

Previous studies sought to identify radiomics features that were stable across influences on image quality resulting from the use of different scanner systems and acquisition and reconstruction methods [9, 12]. Here, we sought to identify image quality that improves the yield of stable and reliable radiomics features. We independently assessed four reconstruction algorithms for radiomics feature extraction, and our investigation encompassed the entire imaging chain including raw data acquisition. Our study thus differed from previous work, in which an image conversion filter was applied to reconstructed image data [22, 23]. The phantom we used had the advantage of featuring complex textures similar to human tissues, enabling us to evaluate feature stability and discriminative power in a realistic setting. Our results confirm limitations in the use of many features in conjunction with FBP and iterative reconstruction but also reveal novel opportunities with deep learning reconstruction that may be considered in retrospective data collection and future protocol implementations for radiomics [26]. Moreover, our results underline that the integration of deep learning into image processing has high potential to improve radiomics research, supporting conclusions from previous reproducibility studies [22, 23].

The limitations of this study include that our results apply only to abdominal imaging with the scanner system, acquisition settings, and reconstruction methods used here. In particular, advantages of deep learning reconstruction remain to be confirmed for implementations by other manufacturers. We analyzed a single phantom and eight biologically equivalent variants of three tissue classes to ensure comparability of our results. However, we cannot provide evidence that results also apply in other tissues or patients. Our assessment of radiomics features involved a thorough characterization in terms of discriminative power and stability. However, we did not investigate feature redundancies, which may reduce the number of suitable features [9]. Also, preprocessing was reported to improve the stability of radiomics features and may be investigated in the future for further increasing feature yield [27].

In conclusion, image quality of CT images reconstructed with filtered back projection, hybrid iterative reconstruction, and model-based iterative reconstruction is inadequate for the majority of radiomics features due to inconsistent tissue characterization, low discriminative power, or low repeatability. Denoising with deep learning reconstruction substantially enhances image quality for radiomics at doses that are typically used clinically. Image reconstruction algorithms can optimize image quality for more reliable quantification of tissues in CT images.

## Supplementary Information

ESM 1  (DOCX 6.65 mb)

## Abbreviations

AiCE: Advanced intelligent Clear-IQ Engine
AIDR 3D: Adaptive iterative dose reduction 3D
CT: Computed tomography
CTDI_vol_: Computed tomography dose index
FBP: Filtered back projection
FIRST: Forward projected model-based Iterative Reconstruction SoluTion
HU: Hounsfield unit
ICC: Intraclass correlation coefficient
OCCC: Overall concordance correlation coefficient

## Declarations

### Guarantor

The scientific guarantor of this publication is Dr. Paul Jahnke.

### Statistics and Biometry

Dr. Florian Michallek and Ulrich Genske have significant statistical expertise.

### Methodology

• prospective

• observational

• performed at one institution
