# Supplementary material for: Deep learning reconstruction improves radiomics feature stability and discriminative power in abdominal CT imaging: a phantom study
Source: Eur Radiol. 2022 Feb 16;32(7):4587–95. doi: 10.1007/s00330-022-08592-y (PMC9213380; doi:10.1007/s00330-022-08592-y)
Supplement: Supplementary file 1 — (DOCX 6.65 mb) [file 330_2022_8592_MOESM1_ESM.docx]

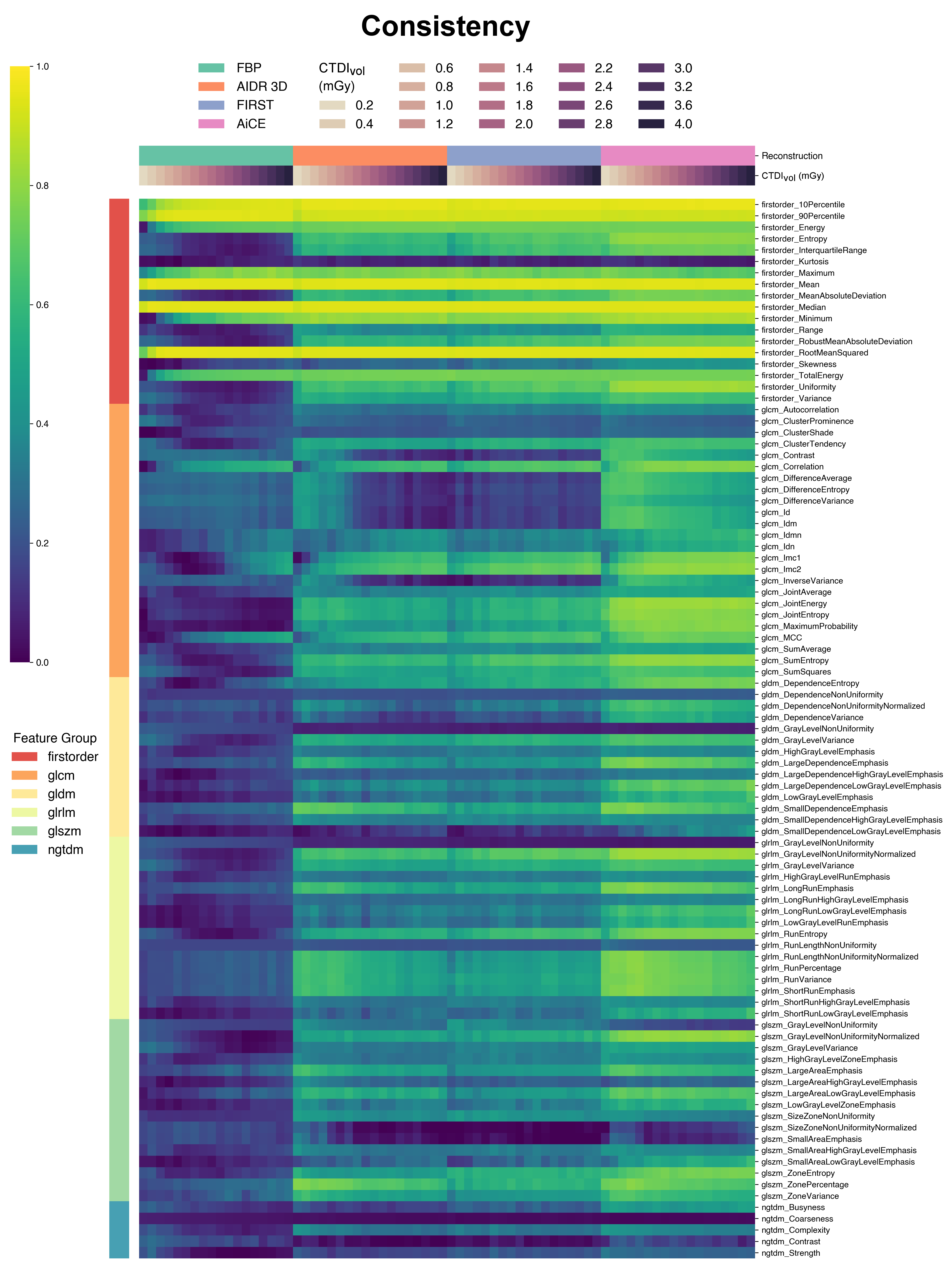


Suppl. fig. 1: Consistency of radiomics features. The heatmap shows the intraclass correlation coefficients (ICCs) of 93 radiomics features per dose and image reconstruction.


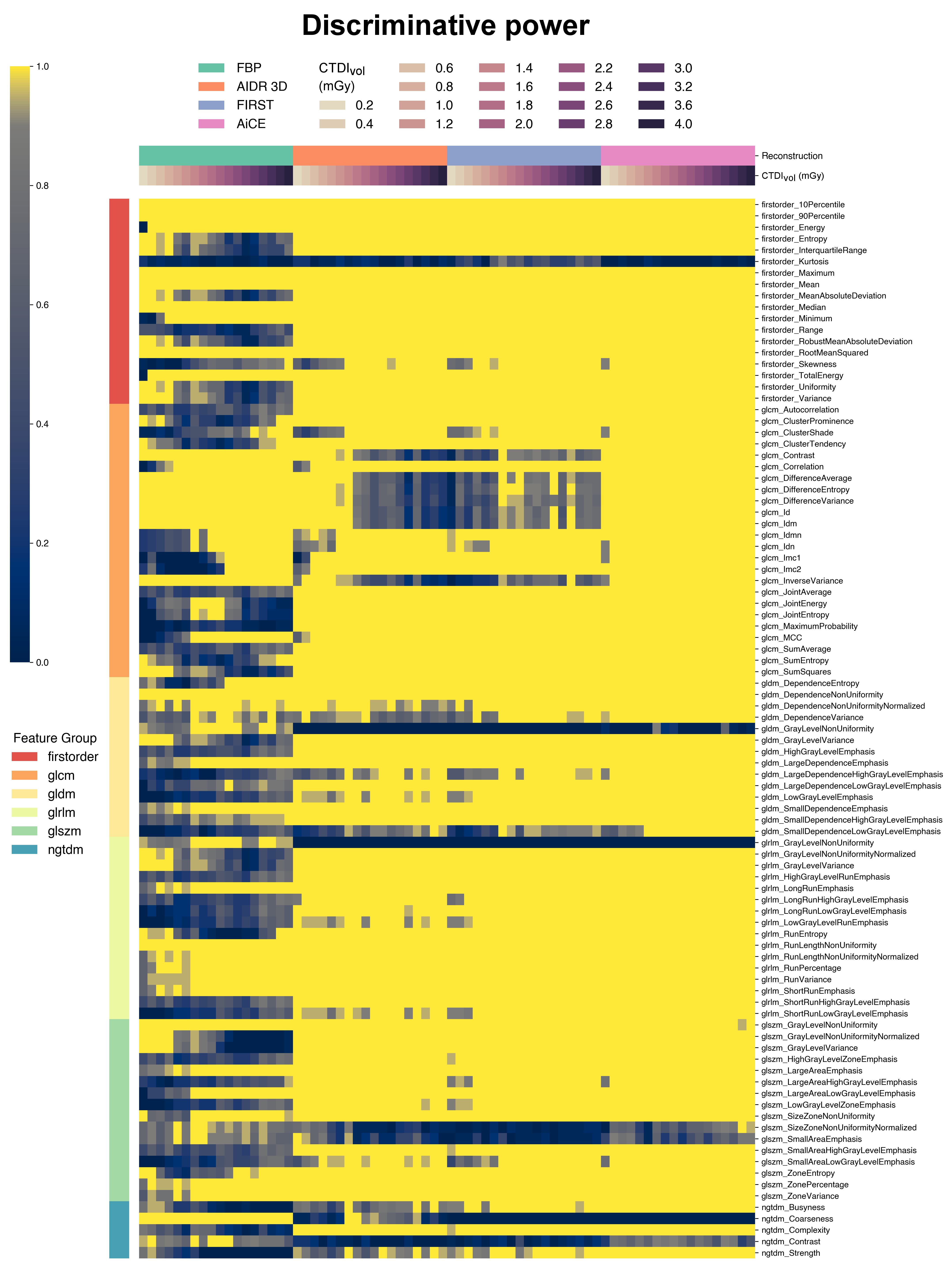


Suppl. fig. 2: Discriminative power of radiomics features. The heatmap shows the fraction of repeated acquisitions for which the p value of the Kruskal-Wallis test was < 0.05 per dose and image reconstruction.


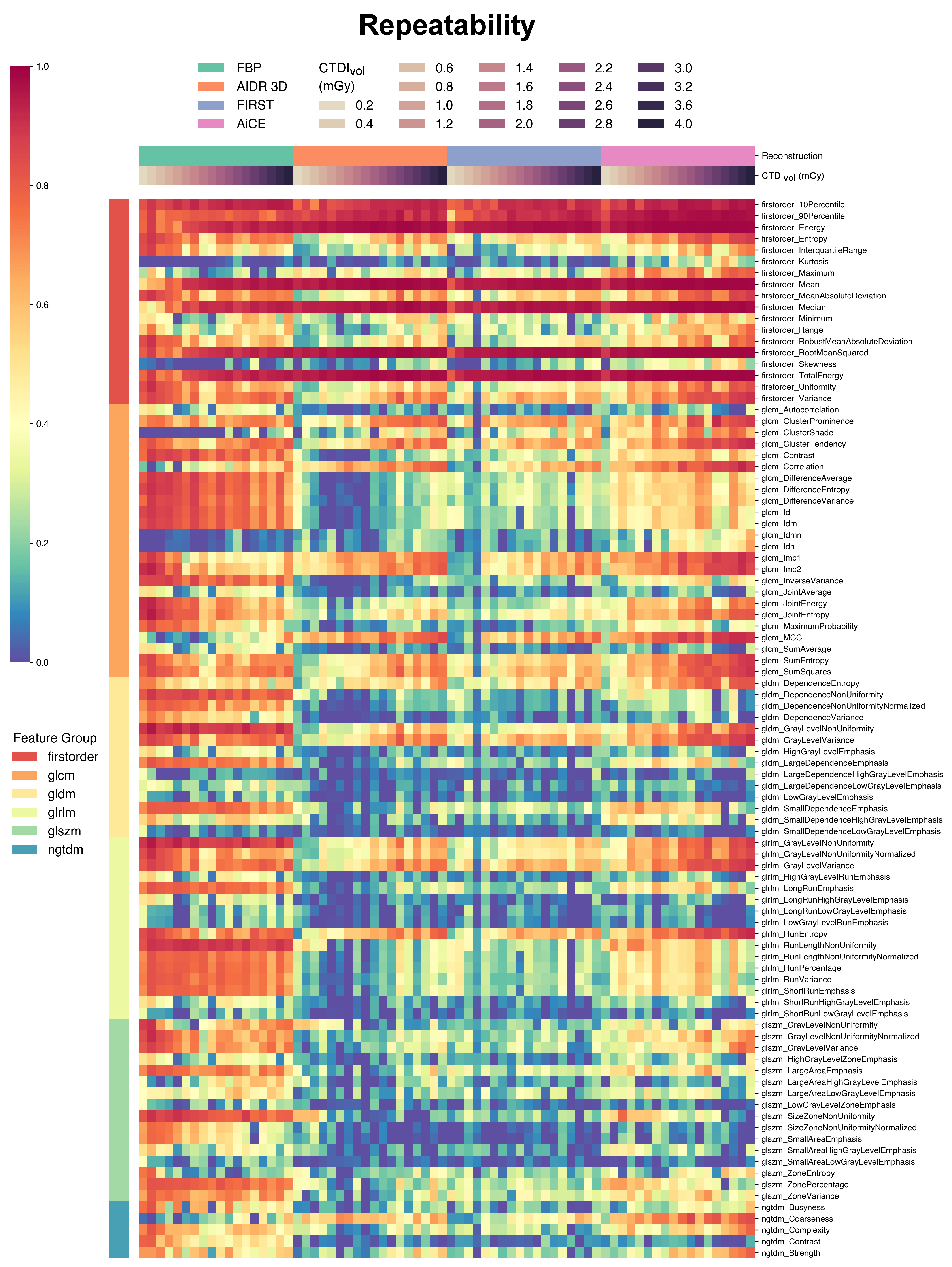


Suppl. fig. 3: Repeatability of radiomics features. The heatmap shows the overall concordance correlation coefficients (OCCCs) of 93 radiomics features per dose and image reconstruction.
